# A Clinicopathological Survey of Basal Cell Carcinoma in an Iranian Population

**Published:** 2013-12

**Authors:** M Zargaran, A Moghimbeigi, AR Monsef, H Teimourian, S Shojaei

**Affiliations:** a Dental Research Center, Dept. of Oral and Maxillofacial Pathology, School of Dentistry, Hamadan University of Medical Sciences, Hamadan, Iran.; b Dept. of Statistics and Epidemiology, School of Public Health, Hamadan University of Medical Sciences, Hamadan, Iran.; c Dept. of Pathology, Hamadan University of Medical Sciences, Hamadan, Iran.; d Dept. of Restorative Dentistry, School of Dentistry, Ghazvin University of Medical Sciences, Ghazvin, Iran.; e Dept. of Oral and Maxillofacial Pathology, School of Dentistry, Hamadan University of Medical Sciences, Hamadan, Iran

**Keywords:** Basal cell carcinoma, BCC, Skin Cancer, Frequency

## Abstract

**Statement of Problem:** Basal cell carcinoma (BCC), the most common skin cancer, is a locally invasive malignant epidermal tumor with ulceration and destruction of underlying structures.

**Purpose:** The purpose of this study was clinicopathological evaluation of BCC in the state and the private pathology centers in Hamadan province during 1990-2010.

**Materials and Method: **In this retrospective study all histopathologically proven cases of BCC were reviewed and the related information including age, gender, place of residency and number of tumors for the patients alongside with the site of occurrence, size, histopathological and clinical type of the lesions were collected and then statistically analyzed, using SPSS software.

**Results:** A total of 804 incidents of BCC were diagnosed in 746 patients (296 females and 450 males) with the most affected site being in the head-face (84.8%), neck (2.6%), trunk (1.6%) and limbs (0.9 %) and 10.1% cases with unknown site. The mean age for the patients was 61.77±13.75 years (63.07± 13.44 for males, 59.81 ± 14.01 for females) and the highest frequency (27.2%) occurred among 60–69 years age group. Nodular type was the most common clinical and histopathological BCC lesions studied whereas the mean size of the lesions was 15.67 ± 11.06 mm with more frequency rate in urban than rural regions.

**Conclusion:** This study focuses on the survey of BCC in Hamadan province but regarding to insufficiency of the data collected by state and provincial pathology centers about the BCC cases reported; it is highly recommended to apply comprehensive questionnaire, which are designated by skillful professionals who are familiar with the lesion nationally.

## Introduction

BCC is the most common skin cancer in human being which statistically constitutes 70%-75% of all skin cancers [1]. This malignant epithelial neoplasm arises from skin basal cells layer and it is mainly caused by chronic exposure to ultraviolet radiation (UV) of sunlight [1-5]. 

Therefore, BCCs occur mostly in those areas of body which are exposed to the sun ray, particularly in the head and neck (80% of the cases) [1, 3, 6-8]. 

This tumor is more diagnosed among adults who are elder than 50 years old [2-3, 5]. BBC is a low-grade malignancy with slow growing, extremely rare metastasis and low mortality rate [1, 5]. It is an expensive and a serious public health problem globally related to its local invasive characteristic since it can cause extensive tissue destruction and considerable disabilities if it is diagnosed and treated with delay [2, 5-6, 9-13].

This matter highlights the importance of public health workers awareness about BCC epidemiological and clinical characteristics. Since the early detection and treatment of lesions is crucial to decrease the functional and cosmetic morbidity and costs [5, 14-15].

Due to the importance of this subject and the rareness of data in Hamadan province; the aim of this study was to survey the clinicopathological characteristics of BCC in state and private pathology centers of Hamadan province during 1990 - 2010.

## Materials and Method

The materials that served as the basis for this retrospective descriptive cross sectional study were collected from the pathology diagnostic centers located in Hamadan province (including 15 state and 4 private pathology centers) during 1990-2010. 

All the reports recorded in this 20- year period were observed and 746 histopathologically proven cases of BCC were found among 142865 reports.

The data of age, gender, occupation, and patients’ residence place, anatomical area of tumor, clinical type, histopathological type and tumor size were extracted from reports. The incomplete information was completed through the clinical files or the histopathological requesting form of tumor. To keep the privacy of patients, all the information were recorded anonymously. Having coded and recorded the collected data; they were analyzed by SPSS software, version 15. To analyze the results, we used descriptive statistics such as mean, standard deviation, frequency tables and also we performed the analytical tests of ***t***-test, Chi-squared and Fisher’s exact test. 

## Results

A total of 804 histopathologically confirmed BCCs were recorded in 746 patients including 712, 28, 8 and 3 patients with 1, 2, 3 and 4 lesions, respectively.

Of these 746 patients, 450 were men (60.4%) and 296 were women (39.6%). The mean age of these patients was (61.77± 13.75); (63.07± 13.44) and (59.81± 14.01) years for men and women respectively with a significant difference between the two groups (Data were analyzed by*** t***-test, *p*= 0.0002). The patients were also categorized with respect to the age range distribution of BCCs, whereas the highest and the lowest frequencies were found among 60-69 (27.2%) and < 19 (0.3%) years age groups, respectively. 

Regarding the variable of gender; for men the highest frequency was among 60- 69 years old (126 cases, 28%) and the lowest was among < 19 years old (2 cases, 0.4%) age groups. Likewise, for women the highest frequency was among 50-59 and 60-69 years old (each 77 cases, 26%) while no cases were found among < 19 years old.

Of all 746 patients contained 134 (18%) urban residents, 63 (8.4%) rural residents and 549 (73.6%) with no record of residence place. 

The occupation status was not recorded for 692 (92.8%) cases and the rest of the patients were farmers (22 cases, 2.9%), housekeepers (16 cases, 2.1%), retirees (9 cases, 1.2%), clerks (3 cases and 0.4%), drivers (3 cases and 0.4%) and plumber (1 case, 0.1%) 

Tumor sizes were recorded in all 804 cases with mean of 15.67±11.06mm. The mean size of 13.97± 10.30 mm was registered for 310 cases of BCCs occurred in females and the mean size of 16.74±11.40 mm was recorded for 494 cases occurred in males, showing a significant difference between male and female groups (Data were evaluated by using*** t***-Test, *p*= 0.001). The smallest and the largest tumor size were 2mm and 100 mm in diameter, respectively. 

Considering anatomical distribution of the total 804 BCCs; 84.8%, 2.6%, 1.6%, and 0.9% of the lesions were located in the area of head-face, neck, trunk, and limbs, respectively. For 81 cases (10.1%), location of the lesions was unknown. The details for anatomical distribution of BCCs with a known site (723 cases) are presented by gender in table 1 while no significant dif ference was observed between the male and female cases (Fisher’s exact test was used for the statistical analys is, (*p*= 0.091).

**Table 1:** Anatomical distribution of BCC in body regarding the gender

**Total** **n(%)**	**Anatomical distribution**				**Gender**
	**Head-face** **n(%)**	**Neck** **n(%)**	**Trunk** **n(%)**	**Limbs** **n(%)**	
275 (100)	266 (96.7)	4 (1.5)	2 (0.7)	3 (1.1)	Female n(%)
448 (100)	416 (92.9)	17 (3.8)	11 (2.5)	4 (0.9)	Male n(%)
723 (100)	682 (94.3)	21 (2.9)	13 (1.8)	7 (1)	Total n(%)

Fisher’s exact test, *p* = 0.091

**Table 2:** Anatomical distribution of BCC in head-face area by gender

**Total** **n(%)**	**Anatomical distribution**									**Gender**
	**Scalp** **n(%)**	**Ears** **n(%)**	**Eyelids and** **peri-orbital n(%)**	**Nose** **n(%)**	**Nasolabial fold** **n(%)**	**Lips and perioral** **region n(%)**	**Forehead** **n(%)**	**Chin** **n(%)**	**Cheek** **n(%)**	
266 (100)	29 (10.90)	8 (3.01)	20 (7.52)	105 (39.47)	12 (4.51)	10 (3.76)	18 (6.77)	2 (0.75)	62 (23.31)	Female n(%)
416 (100)	79 (18.99)	43 (10.33)	34 (8.17)	151 (36.30)	3 (0.72)	7 (1.68)	19 (4.57)	1 (0.24)	79 (18.99)	Male n(%)
682 (100)	108 (15.84)	51 (7.48)	54 (7.92)	256 (37.54)	15 (2.20)	17 (2.49)	37 (5.43)	3 (0.44)	141 (20.67)	Total n(%)

Classified frequencies of BCCs in the head-face area showed the highest and the lowest for nose (37.54%) and chin (0.44%), respectively. Table 2 represents the frequency of lesions in the head-face area by gender.

Respectively nodular and pigmented BCCs had the highest (71%) and lowest (0.2%) clinical presentations, compared to other BCC types including ulcerative (21.9%) and plaque type (0.9 %); while 6% of the lesions were not clinically recorded. Gender-specific frequency of BCCs with known clinical type (756 cases) is shown in table 3, whereas no significant statistical difference was found between men and women (The statistical analysis was performed with Fisher’s exact test, *p*= 0.103).

In 326 of the studied BCCs (40.5%), the histopathological types were not documented. Among 478 cases of the lesions with known histopathological types; nodular (331 cases, 41.2%) and baso-squamous (4 cases, 0.5%) were the most and the least frequent ones respectively. Whereas the rest of the diagnosed tumors were adenoid (6.3%), pigmented (4%), morpheaform (3.2%), superficial (2.4%), and keratotic (1.9%). Gender-specific frequency for the diagnosed histopathological types of the studied BCCs is presented in table 4, showing a significant statistical difference between males and females (The data were analyzed using Chi-squared test, *p*= 0.047).

## Discussion

This retrospective study analyzed and reported clinicopathological characteristics of BCCs (diagnosed in the state-owned and private pathology centers) in Hamadan province as follows:

Regarding to the results obtained in this study, 60.4% and 39.6% of BCCs occurred in males and females respectively; which is almost similar to the findings of Lotfinejad et al. (67.4% men and 32.6% women) [16] . It seems the frequency of BCCs is higher among men rather than women, reflecting the differences in the nature of men and women activities that was also approved by previous studies including Hakverdi et al. [5], Revenga Arranz [17], Hakimi [18], Bastiaens et al. [19], and Raasch et al. [20].

Ultraviolet radiation (UV) is known as the main risk factor BCCs [5, 21] which passes throughout the atmosphere and reaches to the earth surface in more quantities due to destruction of the ozone layer nowadays [22].

Therefore, over does and long time exposure to solar UV can increase the risk of occurrence of this type of malignancy especially due to job conditions and outdoor activities. Nevertheless, Flohil et al. [23], Custódio et al [1], Omari et al. [24], and Bariani et al. [22] reported more cases of these tumors in women than men which might be resulted by other risk factors including exposure to chemical carcinogens, HPV infection (possibly), type of skin, chronic irritation, chronic inflammation, burns, skin lesions, immunological and genetic factors apart from the UV exposure [5].

**Table 3:** Frequencies of different clinical types of BCC regarding gender

**Total** **n(%)**	**Clinical type**				**Gender**
	**Nodular type ** **n(%)**	**Ulcerative type** **n(%)**	**Pigmented type ** **n(%)**	**Plaque type ** **n(%)**	
290 (100)	225 (77.6)	60 (20.7)	0 (0)	5 (1.7)	Female n(%)
466 (100)	346 (74.2)	116 (24.9)	2 (0.4)	2 (0.4)	Male n(%)
756 (100)	571 (75.5)	176 (23.3)	2 (0.3)	7 (0.9)	Total n(%)

Fisher’s exact test, *p*=0.103

**Table 4:** Frequencies of histopathological types of BCC regarding gender

**Total** **n(%)**	**Histopathologic type**							**Gender**
	**Morpheaform** **n(%)**	**Nodular** **n(%)**	**Superficial** **n(%)**	**Adenoid** **n(%)**	**Pigmented** **n(%)**	**Keratotic** **n(%)**	**Basosquamous** **n(%)**	
180 (100)	14 (7.8)	120 (66.7)	13 (7.2)	16 (8.9)	12 (6.7)	4 (2.2)	1 (6)	Female n(%)
298 (100)	12 (4)	211 (70.8)	6 (2)	35 (11.7)	20 (6.7)	11 (3.7)	3 (1)	Male n(%)
478 (100)	26 (5.4)	331 (69.2)	19 (4)	51 (10.7)	32 (6.7)	15 (3.1)	4 (0.8)	Total n(%)

Chi-squared test, *p*= 0.047

In industrialized societies, people are more likely to be exposed to artificial sources of ultraviolet and ionized radiation [16]. In recent years with considering the equal job opportunities; women are also in danger of being exposed to artificial sources of UV. Therefore, more cases of BCCs among women in such societies are explicable. 

However, racial and cultural factors under various geographical regions may influence the frequencies of BCC cases occurring in men and women. Moreover, the related prevention programs which might be adapted based on the above mention factors would influence the frequency of this disease.

Ageing is considered as another risk factor for BCC [22]. BCC cases more often occur among adults and particularly old people (older than 50) because this population is assumed to have been more exposed to the sunrays and its cumulative effects which lead to direct damage of DNA [5, 25]. 

On the other hand, the immune system of body becomes less efficient and DNA regeneration capacity decreases with aging; which consequently results in higher possibility of BCC development [22].

The mean age of patients in this study was (61.77± 13.75) including (63.07± 13.44) for men and (59.81± 14.01) for women, almost similar to the results reported by Hakverdi et al. [5], Scrivener et al. [26], Custódio et al. [1], Bariani et al. [22], Toosi et al. [15], Meamar et al. [21], and AliAhiaee [27]. However, the mean age of the patients (71.40±11.24) and the gender-specific mean age (70.30±13.9 for men, and 72.30±12.70 for women) reported by Revenga Arranz et al. [17] were considerably higher compared to the current study. 

Furthermore, statistical surveys revealed that the difference between mean ages of men and women is significant in our study, similar to the study performed by Meamar et al. [21].

Minimum and maximum ages of the patients were reported as 6 and 107 years respectively, by Scrivener et al. [26] that are close to the minimum and maximum ages (2 and 107 years) reported in the present study. Anyway, they didn’t discuss about BCC occurrences among children in particular [26]. 

BCC generally occurs in people who are elder than 50 years old [5]. However, its childhood occurrence is rare [28-29] and is usually associated with predisposing genetic disorders such as nevoid basal cell syndrome, Bazex syndrome, sebaceous nevus, albinism, Rombo syndrome and xeroderma pigmentosum. Sometimes Childhood onset of BCC might be due to the radiotherapy as well [28-30].

 It is well known that skin cancer may also arise at those body sites with a history of radiotherapy even if there is no evidence of chronic radiation damage like atrophy , irregular hyperpigmentation and scar [31] . Relative risk of BCC is higher for the irradiated children who have been gone through radiotherapy because of an enlarged thymous gland and tinea capitis [31]. Radiotherapy for medulloblastoma, the most common malignant brain tumor in children, might cause subsequent BCC in the irradiated skin area [32].

Moreover, it needs to be mention that Idiopathic childhood onset of BCC is much less common [28].

There was no detailed information on the clinical documents of the only 2-year old patient for detecting the possible effective causes related to his BCC in this study. Therefore, evaluation of any predisposing condition among children by subspecialists and pediatric caregivers is highly recommended.

These values in the studies by Bariani et al. [22], Revenga Arranz et al. [17] and Toosi et al. [15] were 26 - 90 years, 28 - 98 years, and 26 - 86 years, respectively. 

According to the obtained results; the highest frequency of BCCs was observed in 60- 69 years age group (27.2%) and the lowest frequency was seen among < 19 years old (3%). Likewise, Meamar et al. [21], Omari et al. [24], Hakimi [18], and AliAhiaee [27] have also reported the age peak of BCC in the 60's but in the study by Bariani et al. [22] it was in the 70's. 

The lowest age group of BCC patients reported by Meamar et al. [21] , Custódio et al. [1] AliAhiaee [27] and Hakimi [18] were < 15 , < 20 , 20-29 and <40 years old, respectively. It seems that the differences among the defined age ranges in different studies might have influenced the lowest age groups of BCC patients. 

In this study, occupations of 54 patients were recorded, among which farming with 22 cases and housekeeping with 16 cases were the most common. Similarly, farming (-ranching) and housekeeping were the most common jobs in the studies by Hakimi [18] and AliAhiaee [27] ,although in AliAhiaee [27] study farming was in second order after housekeeping. Meamar et al. [21] reported housekeeping as the most common job for the BCC patients.

Although BCC is more expected to happen among outdoor occupations, other aforementioned risk factors should not be underestimated. It also should be mentioned that some housekeeping activities go on outdoor where there is the possibility of sunrays exposure [18]. 

Our results, regarding the place of residence of the patients, revealed that among 197 cases with known places of residence, 68.02% were from urban and 31.98% were from rural regions. Similar to the findings of this study; there were reports on more cases of BCC among urban residents than rural population [27, 33-34]. 

Even though agricultural activities was found to be the main occupation for most of the BCC patients; which elusively explain more BCC cases among rural people, it should be mentioned that type of occupation (92.8%) and place of residency (73.6%) was not recorded for most of the patients in this study. on the other hand, urban population usually have more access to health care centers, therefore, resulting in more registered cases of BCC in urban area [27]. Additionally, places of occupation and residency for some cases may not be the same as there are many urban residents who are working in agricultural sectors [27].Compared to our findings, the results reported by Hakimi [18] on more cases of BCC among residents of rural (60.78%) rather than urban region (39.13%) seems a contradiction.

Concerning the previous studies; occurrences of BCC cases can be solitary or multiple [5, 17, 22, 26] as in this study for 712 (95.44%), 28 (3.75%), 8 (1.07%), and 3 (0.4%) patients number of the lesions were 1, 2, 3, and 4, respectively. This means occurrence of 804 BCC lesions among 746 patients. Similar situation was reported in other studies [5, 15, 17-18, 22, 26]; for instance Bariani et al. [22] found a total number of 253 lesions among 202 patients containing 82.2% , 12.4% and 4% with 1, 2 and 3 ≤ lesions, respectively. Revenga Arranz et al. [17] also found a total number of 509 lesions among 413 patients including 1, 2, 3, 4, 5, and 6 lesions in 84.7%, 10.6%, 3.1%, 0.7%, 0.2%, and 0.4% of patients, respectively. Moreover, Hakimi [18] approved single and multiple BCC lesions among 53 (76.81%) and 16 (23.18%) of the studied patients. 

According to previous studies, some factors that may increase risk of multiple BCCs can be listed as follows:

- Conditions that lead to decrease immunity (immunosuppressed states) such as transplant recipients [19].

- Radiotherapy [31].
- Immuno-genetic basis including cases of HLA-DR4 [3].
- Syndromes like Nevoid basal cell syndrome, Bazex syndrome [3].
- Arsenic treatment in psoriasis patients [35].
- Cases of genetic polymorphisms in CYP2D6 [36].
- Patients with BCC of the trunk [37].

Ironically, no cases of multiple BCCs in the trunk were found in this study whereas all the multiple cases were in head-face or neck areas. No information about other mentioned factors has been recorded in many documents of the BCC patients.

Despite the fact that metastasis is rarely seen in BCC, it is known that the lesion size might be considered as a risk factor for estimating the possibility of metastasis occurrence since the likelihood of metastasis in tumors with diameters larger than 3cm, larger than 5cm, and larger than 10cm have been estimated as much as 1-2%, 20-25%, and more than 50%, respectively [5]. However, no metastasis was observed among the cases in this study. 

 Different studies [1, 15, 18, 22] offered different classifications for BCC sizes whereas in this study the lesions^’^ mean size was considered based on the study of Toosi et al. [15].

The lesion mean size was 15.67±11.06 mm with a minimum and maximum of 2mm and 100mm, respectively, almost similar to the mean size of 15±8.8 mm reported by Toosi et al. [15]; although the minimum (3mm) and maximum (65mm) sizes were different.

Mean size of the lesion among men and women were 16.74± 11.40 mm and 13.97±10.30mm, respectively, showing a significant difference at *p* = 0.001.

The cause of such difference might be explained through more sensitivity and attention of women to their health and any unusual changes in their body, especially in the head-face and neck areas; which persuade them to visit a doctor more frequently and sooner when compared to men.

Most of the skin cancers occur in those parts of the body where are more likely to be exposed to the sunlight [1]. In these parts of the body, especially in the head-neck area, BCC lesions seem to be more frequent when compared to other parts with complete or relative coverage [1, 5, 22]. 

Anatomical distribution of the BCC lesion ,in descending order, were for head- face , neck, trunk and limbs which is in agreement with the findings of Hakverdi et al. [5], Omari et al. [24] and Toosi et al. [15]. 

In the head-face area the highest frequencies of the lesion were for nose, cheek, and scalp, respectively; while the lowest frequency was for chin. Similarly, nose is reported as the most susceptible site for BCC by Custódio et al. [1], Hakverdi et al. [5], Meamar et al. [21], Hakimi [18], Lotfinejad et al. [16], and AliAhiaee [27]. Same as this study, AliAhiaee [27] reported cheek as the second, Lotfinejad et al. [16] mentioned scalp as the third, and Hakimi [18] asserted chin as the lowest susceptible sites for the lesion^’^s occurrence in the head-face area. However, findings ‌by Toosi et al. [15] were not in agreement with our results. 

In this study nodular and ulcerative (71% and 21.8% respectively) forms composed the most common clinical types of the BCC lesions .The pigmented types (0.2%) were the least common and clinical type for 5.97% of the lesions were unclear. According to Toosi et al. [15], the most common clinical types were nodulo-ulcerative (47.7%) and nodular (31.6%) while the ulcerative (0%) ones were among the least commons. 

In Hussain et al. [38] study nodulo-ulcerative (36.7%) and nodular (33.3%) ones were the first and second most common clinical types of the lesion, respectively, whereas, unlike to our study, pigmented types (30%) were the third most common among the BCCs reported.

Apparently, histopathological type of the BCC tumor is one of the factors that play an important role during treatment planning. Hence, pathologists remark the histopathological type when report BCC cases [5]. The most common reported histopathological type of BCC is nodular ones [5, 15, 19-20, 22, 26, 38-39] which is in agreement with our results. Anyway, there are differences regarding the least common histopathological types according to various studies as in Bariani et al. [22] study pigmented type, in Hakverdi et al. [5] and Scrivener et al. [26] studies morpheaform type, and in Hussain et al. [38] study morpheaform and cystic types reported to be the least common histopathological types of the BCC lesions.

In current study baso-squamous type is the least common histopathological type of BCC, similar to the report by Toosi et al. [15]. 

## Conclusion

This study evaluated the frequency as well as the clinical and the histopathological characteristics of BCC cases, recorded in Hamadan province, alongside with the interrelations of various effective variables on the lesion compared to other geographical regions.

 However, like previous studies, insufficient data regarding the information of skin phenotype, rate of exposure to sunshine, physical and chemical protective usages, etc. made it difficult to obtain accurate reports and reach clear reasons for the studied cases.

Therefore, it is recommended to revise current methods of data collection by training the staffs of healthcare system including dentists, doctors and other healthcare workers to increase their knowledge on proper recording of the information of the observed lesions. Additionally, improving public awareness about people who are most at risk could facilitate screening of BCC lesion throughout the society. Furthermore, it is necessary to collect data of observed BCC cases, using comprehensive questionnaires; which are designated by skillful professionals who are familiar to the lesion seems necessary.

Regarding the recent developments in the software programs and information technology to record patients’ information, it deserves to use such technologies and equipments in order to save patients accurate information as digital files. This could be a great help to evaluate current situation of BCC lesion and prediction of its behavior through the society in the study area and then conduct every appropriate groundwork including taking positive steps to promote health cares for this lesion and subsequently reducing the incidence of the disease in the society.
